# Rhanteriol, a New *Rhanterium suaveolens* Desf. Lignan with Pharmacological Potential as an Inhibitor of Enzymes Involved in Neurodegeneration and Type 2 Diabetes

**DOI:** 10.3390/plants12020301

**Published:** 2023-01-09

**Authors:** Soumia Belaabed, Ayoub Khalfaoui, Valentina Parisi, Valentina Santoro, Daniela Russo, Maria Ponticelli, Magnus Monné, Khellaf Rebbas, Luigi Milella, Giuliana Donadio

**Affiliations:** 1 Department of Chemistry, Research Unit, Development of Natural Resources, Bioactive Molecules, Physicochemical and Biological Analysis, University Mentouri, Route Ain ElBey, Constantine 25000, Algeria; 2 Dipartimento di Farmacia, Università Degli Studi di Salerno, via Giovanni Paolo II 132, Fisciano, 84084 Salerno, Italy; 3 PhD Program in Drug Discovery and Development, Department of Pharmacy, University of Salerno, Via Giovanni Paolo II 132, Fisciano, 84084 Salerno, Italy; 4 Dipartimento di Scienze, Università Degli Studi Della Basilicata, Viale dell’Ateneo Lucano 10, 85100 Potenza, Italy; 5 BioActiPlant s.r.l., Viale Dell’Ateneo Lucano 10, 85100 Potenza, Italy; 6 Natural and Life Sciences Department, Mohamed Boudiaf University, M’Sila 28000, Algeria

**Keywords:** *Rhanterium suaveolens* Desf., rhanteriol 1, NMR, *α*-amylase inhibition, *α*-glucosidase inhibition, cholinesterases inhibition, molecular docking

## Abstract

Several specialized plant metabolites are reported to be enzyme inhibitors. In this investigation, the phytochemical composition and the biological activity of *Rhanterium suaveolens* Desf. were studied. One new lignan (rhanteriol 1) and seven known secondary metabolites were isolated from the aerial parts of *R. suaveolens* by using different chromatographic procedures. The biological properties of the *R. suaveolens* extracts and the new compound were evaluated by measuring their ability to inhibit the cholinesterase and carbohydrate-hydrolyzing enzymes, using cell-free in vitro methods. The new lignan, rhanteriol, was shown to inhibit *α*-amylase and *α*-glucosidase (IC_50_ = 46.42 *±* 3.25 μM and 26.76 ± 3.29 μM, respectively), as well as butyrylcholinesterase (IC_50_ = 10.41 ± 0.03 μM), with an effect comparable to that of the respective standards, acarbose and galantamine. Furthermore, docking studies were performed suggesting the interaction mode of rhanteriol with the active sites of the investigated enzymes. The obtained data demonstrated that the aerial part of *R. suaveolens* could represent a source of active molecules, such as rhanteriol, usable in the development of treatments for preventing or treating type 2 diabetes mellitus and neurodegeneration.

## 1. Introduction

Plants play an important role in discovering natural products with therapeutic effects offering special features compared to synthetic molecules [1]. Enzyme-inhibitory agents are gaining attention due to their application against several ailments, since enzyme inhibition has been recognized as an alternative and significant target [2]. Specifically, inhibition of *α*-amylase and *α*-glucosidase enzymes could represent an important strategy for treating diabetes and/or obesity, since natural inhibitors of these enzymes can delay the uptake of dietary carbohydrates and reduce postprandial hyperglycemia [3,4]. In the same way, plant cholinesterase-inhibitors could be a therapeutic or complementary approach for neurodegenerative disorders [5]. From this perspective, computer-based methods such as molecular docking may represent an attractive tool for structure-based drug design and screening of high-quality enzyme inhibitors from herbs [6].

Several plants’ specialized metabolites, such as lignans [7], flavonoids [8,9], and quinic acid derivatives [10], are reported as enzyme inhibitors. Specifically, Tunisian flora is known to be characterized by several medicinal and aromatic species producing active metabolites with multiple biological activities. Of particular interest are bioactive molecules from plants belonging to the genus *Rhanterium* (Asteraceae)*,* which are mainly distributed in the Arabian Peninsula, western North Africa (Tunisia, Algeria, and Morocco), Iran, and Iraq. To this genus has been attributed in the literature only three species: *R. adpressum* Coss. & Durieu, *R. suaveolens* Desf., and *R. epapposum* Oliver. Moreover, a hybrid between *R. adpressum* and *R. suaveolens,* the *R. intermedium* Coss. & Durieu ex Pomel, has also been accepted as a fourth species. In this investigation, the attention was focused on *R. suaveolens,* a forage plant commonly known as ″Arfasj″, grazed on by camel and sheep in the desert and used by locals in the process of cheese production and as an antidiuretic in folk medicine [11]. However, only a few pieces of information are present in the literature about this species. It seems that the methanolic extract from the aerial part of *R. suaveolens* from Tunisia reported antioxidant, anti-inflammatory, and anti-tyrosinase activities [12] attributable to the presence of flavonols (glycoside derivatives of quercetin and kaempferol) and hydroxycinnamic acids (chlorogenic acid, caffeic acid, and their derivatives) [12,13]. Furthermore, a previous investigation into *R. suaveolens* phenolic composition revealed the presence of coumarins (scopoline, fraxetin, and scopoletin), polyacetileneic alcohols, nor-isoprenoid glucoside (ranthenone glucoside), ceramides, and steroids (sitosterol-3*β*-*O*-[6′-palmitoyl-*β*-d*-*glucopyranoside]) with antimicrobial activity [14,15]. The action against microbes, together with an anti-cholinesterase activity, was also demonstrated for *R. suaveolens* and *R. epapposum*, and has been attributed to the presence of essential oils (spathulenol, carvacrol, caryophyllene oxide, linalool, *α*-terpineol, *α*-terpinolene, *β*-cadinol, pinocarvone, and perillaldehyde) [16,17,18]. Based on this background, this investigation aimed to provide a phytochemical analysis of *R. suaveolens* leaves, together with an analysis of its ability to inhibit enzymes involved in neurodegenerative disorders (acetylcholinesterase and butyrylcholinesterase) and, for the first time, its ability to affect type 2 diabetes mellitus (*α*-amylase and *α*-glucosidase). The chemical investigation led to identify one new lignan (**1**), and seven known components: one lignan (**2**), two phenolic acids (**3**-**4**), two flavonoids (**5**-**6)** and two quinic acid derivatives (**7**-**8**). Moreover, within the framework of a project aimed at studying new enzyme inhibitors useful for treating type 2 diabetes mellitus and neurodegenerative disorders, a molecular modelling study was performed on the new compound **1**.

## 2. Results

### 2.1. Isolation, Molecular Elucidation, and Bioactivity

The aerial parts of *R. suaveolens* were extracted using solvents of increasing polarity to obtain *n*-hexane, chloroform, and methanol extracts. The methanol extract was dried and subjected to liquid–liquid separation to obtain *n*-butanol (BuOH) and H_2_O fractions. A bioassay-oriented approach evaluating the inhibition of enzymes such as cholinesterases, α-glucosidase, and α-amylase was performed, and the BuOH fraction was found to be a significant inhibitor of the α-amylase, with an IC_50_ value of 1.47 ± 0.3 µg/mL. Moreover, BuOH reported a weak inhibition of cholinesterase enzymes, showing 16% and 10% of inhibition at 100 ug/mL against acetylcholinesterase (AChE) and butyrylcholinesterase (BuChE), respectively. No α-glucosidase inhibition was demonstrated at tested concentrations. 

**Figure 1:**
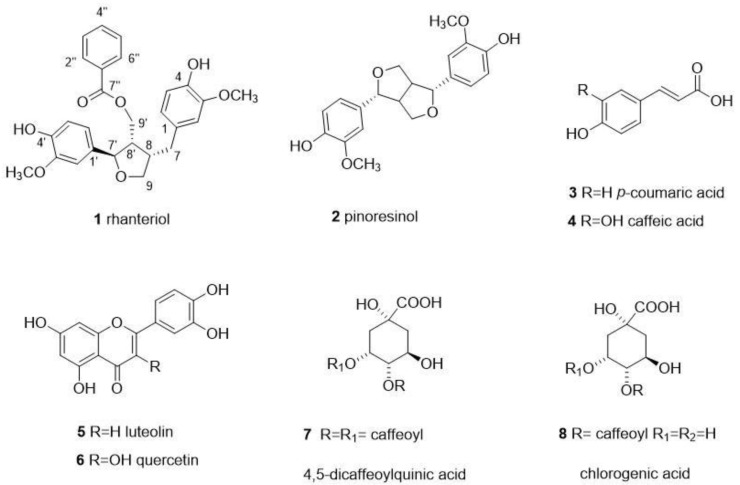
Compounds isolated from *R. suaveolens* methanol extract.

Based on these preliminary and interesting results, the *n*-BuOH extract was subjected to different chromatographic procedures to afford one new (**1**) and seven known (**2**–**8**) compounds (Figure 1). 

Compound **1** was isolated as a yellow amorphous powder. Its molecular formula was determined as C_27_H_28_O_7_ from the protonated and sodiated molecular ions at *m/z* 485.1887 [M+H]^+^, 487.1717 [M+Na]^+^ in its HRESIMS spectrum. Furthermore, from the ^13^C NMR spectrum, 14 index of hydrogens deficiency in molecule **1** were detected. Specifically, the ^13^C-NMR spectrum (Table 1) exhibited 27 signals, which were assigned to three aromatic rings, one ester group (167.8 ppm), two hydroxymethylenes (72.5 and 64.6 ppm), one hydroxymethine (85.4 ppm), two methines (50.5 and 44.3 ppm), one methylene 34.3), and two methoxy groups (56.3 and 56.4). This obtained profile suggested the presence of a lignan derivative [19]. On the other hand, the ^1^H NMR spectrum (Table 1) showed the presence of 11 aromatic protons attributable to two 1,3,4-trisubstituted aromatic rings and one benzoic acid. Homonuclear Correlation Spectroscopy (H-1H COSY) and 1D Total Correlation Spectroscopy (1D-TOCSY) experiments showed correlations between H-2/H-6, H-2′/H-6′, H-2″/H-6″, H-7/H-9′, H-7/H-7′. The connectivity of each proton to the respective carbon was confirmed in the heteronuclear-single-quantum-coherence (HSQC) spectrum, while ^13^C NMR data assignments (Table 1) were made, using a heteronuclear-multiple-bond-correlation (HMBC) experiment. The HMBC correlations between the signal of H-2″, H-6″, and H-9′ and the signal at 167.8 ppm (C-7″) indicated benzoic acid esterification at C-9′. Analysis of the 2D NMR spectra, especially the HMBC data, confirmed that **1** was a lignan with a similar structure to agastinol [19], with the only difference being the presence of benzoic acid which was esterified at C-9′ in **1** instead of *p*-hydroxybenzoic acid in agastinol. Based on the above evidence, the structure of **1** was determined as 4-benzoic acid 4-(4-hydroxy-3-meethoxybenzyl)-2-(4-hydroxy-3-methoxyphenyl) tetrahydrofuran-3-ylmethyl ester, a new natural lignan named rhanteriol (Figures S1–S6). 

**Figure 2:**
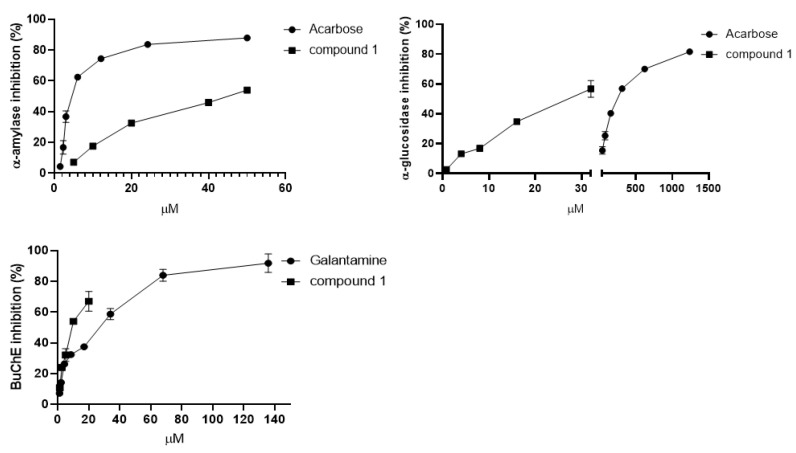
Enzymatic inhibition of compound 1 (rhanteriol) against α-amylase, α-glucosidase and BuChE.

Lignans have important biological properties; previous studies reported the cholinesterase inhibition of lignans of *Citharexylum spinosum* L. [20], *Vitex negundo* L. [21], and other species [7,22,23], along with carbohydrate-hydrolyzing enzymes inhibition [23,24]. For this reason, it was decided to test the bioactivity of the newly isolated lignan. Firstly, the cytotoxicity of **1** was investigated using three human tumor cell lines (HeLa, U937, and Jurkat) and one nontumor human epidermal-keratinocyte line (HaCaT) at 100, 50, 25, µM for 48 h by MTT assay, revealing that compound **1** was not toxic at the tested concentration (data not shown) [25,26]. Hence, the compound **1** inhibitory activity was evaluated against α-amylase and α-glucosidase, the AChE and BuChE enzymes. According to the literature reported on other lignans, the new lignan **1** was able to inhibit both enzymes involved in diabetes, showing IC_50_ values of 46.42 ± 3.25 μM and 26.76 ± 3.29 μM, for α-amylase and α-glucosidase, respectively (Figure 2). These results seem to be promising when compared with acarbose, which was used as a reference drug (IC_50_ 5.65 ± 0.11 μM for α-amylase, IC_50_ 241.32 ± 4.49 μM for α-glucosidase). As regards the anti-cholinesterase activity, surprisingly, the new compound showed a weak activity against the AChE enzyme, with only 21% of inhibition at 100 μM, but a higher inhibition against the BuChE enzyme (IC_50_ 10.41± 0.03 μM), close to that of galantamine, which in this case was used as the standard (IC_50_ 11.63 ± 1.87 μM) (Figure 2). 

### 2.2. In Silico Docking of the New Compound from Rhanterium Suaveolens in the Enzymes 

**Figure 3:**
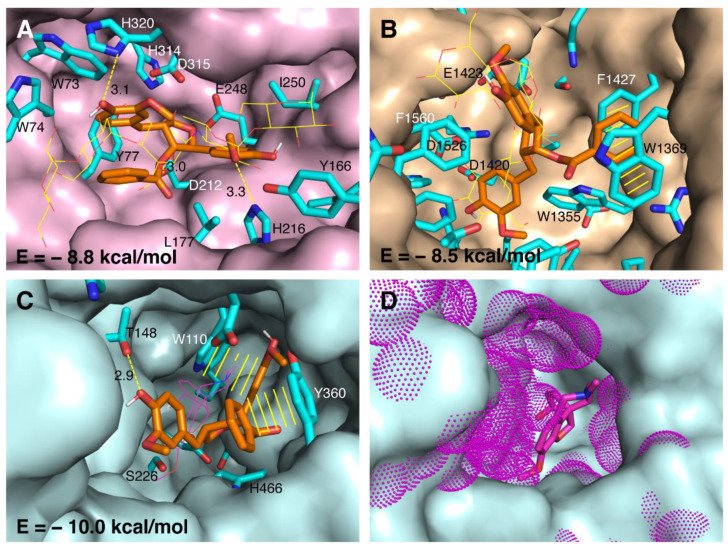
Docking of rhanteriol in the structures of α-amylase, α-glucosidase and in the homology model of BuChE. The binding pockets of α-amylase (pink surface in (**A**)), α-glucosidase (beige surface in (**B**)), BuChE (light cyan surface in (**C**,**D**)) and AChE (additional density with respect to BuChE in magenta-dot surface in (**D**)) with rhanteriol interacting-residues in sticks (cyan carbons). Rhanteriol is shown in sticks (orange carbons), acarbose in lines (yellow carbons) and galantamine in lines/sticks (carbons in magenta). The hydrogen bonds are indicated with dashed yellow lines and the distances in Å, and π-π interactions with solid yellow lines. The binding energies (E) of rhanteriol, obtained by Autodock vina, are indicated for each protein.

Molecular docking of rhanteriol (**1**) was performed in 3D structures of human α-amylase and α-glucosidase, as well as the homology model of BuChE, to investigate further its interactions with these enzymes. In the semi-rigid docking procedure used, rhanteriol (**1**) and the side chains of the residues in the active/inhibitor site pocket are given conformational freedom, whereas the rest of the protein is rigid. Searches of many binding possibilities in the so-called conformational space result in docking solutions for which the theoretical binding-energies are calculated, and the lowest binding-energy (highest affinity) solutions are ranked. The top-ranked docking solutions of rhanteriol are displayed in Figure 3. According to the docking simulation, rhanteriol in the active site of α-amylase (Figure 3A) binds the residues H320, D212, and H216 through hydrogen bonds, and interacts with several other residues. Specifically, it overlaps the central part of the oligosaccharide analogue acarbose, which is a much more hydrophilic inhibitor, and is present in the original structure. The tetrahydrofuran moiety of rhanteriol penetrates deepest into the active site of α-amylase, making contact with the catalytic residues D212 and E248. In contrast, the 4-ethylguaiacol and the benzoate rings are bent outwards in parallel towards one side along the binding cleft and the 4-methylguaiacol, and backwards towards the other side. Rhanteriol in the binding site of α-glucosidase (Figure 3B) does not form any hydrogen bonds, but the benzoate ring of rhanteriol is sandwiched between W1369 and F1427 by π-π interactions. In α-glucosidase, the 4-ethylguaiacol moiety of rhanteriol is deepest in the active groove and, together with tetrahydrofuran and 4-methylguaiacol rings, overlaps with the three hexoses of acarbose that are positioned deeper in the binding pocket. Thereby, the catalytic residues D1420, E1423, and E1526 are completely blocked from substrate access. In BuChE (Figure 3C), the 4-methylguaiacol and tetrahydrofuran moieties of rhanteriol penetrate deep into the binding pocket, leaving the 4-ethylguaiacol and the benzoate in parallel towards the active-site entrance. Rhanteriol binding is stabilized by a hydrogen bond between the 4-ethylguaiacol hydroxyl group and T148, as well as π-π interactions between 4-methylguaiacol and W110 and between the benzoate group and Y360. The tetrahydrofuran group is the closest part of rhanteriol to overlap with the BuChE-inhibitor galantamine, which, although mainly hydrophobic as rhanteriol, is a much smaller and less flexible structure. BuChE’s binding pocket is deeper and much narrower than those of α-amylase and α-glucosidase, and the binding of rhanteriol excludes substrate access to the catalytic triad S226, H466, and E353, which is at the bottom of the cavity. Based on the docking results, the theoretically calculated binding energies of rhanteriol to α-amylase, α-glucosidase, and BuChE, range from −8.5 to −10.0 kcal/mol, and are compatible with high affinities. 

The finding that in the inhibition assays, rhanteriol inhibited BuChE much more effectively than AChE was surprising, as these two proteins have many similarities in structure, catalysis, substrates, and inhibitors. The inability of rhanteriol to inhibit AChE at low concentrations might be explained by the fact that this protein has an even more constricted entrance to the active site pocket than BuChE (Figure 3) and may, therefore, not allow rhanteriol to enter easily.

## 3. Discussion

Nowadays, there is a continuous and strong interest in finding new and alternative therapeutic strategies for managing several pathological conditions such as Alzheimer’s disease and diabetes. Many synthetic drugs are normally used to treat these diseases, but side effects often accompany the beneficial effect. On the other hand, natural products could be a good option, due to their higher safety and multiple biological properties on different sites of action. Several specialized metabolites are known for their antioxidant properties, which are important for reducing oxidative stress, a characteristic condition underlying several diseases [27,28]. In addition, these compounds are also able to interact with key enzymes involved in several disorders. In this study, the activity against enzymes such as cholinesterases, α-glucosidase, and α-amylase of either the aerial parts of *R. suaveolens* BuOH and the H_2_O fraction or compound 1, was investigated. To date, only one scientific article has reported the evaluation of *R. suaveolens* anti-cholinesterase activity [29], while no studies have been carried out regarding the ability to inhibit α-glucosidase and α-amylase. Specifically, the essential oil of *R. suaveolens* was previously demonstrated to be inactive against AChE and BuChE, while the methanolic extract was shown to exert a moderate inhibition against AChE and a good BuChE inhibition (IC_50_ = 168.76 ± 0.62 and 54.79 ± 1.89 µg/mL, respectively) [29]. In this investigation, data from the anti-cholinesterase activity of the BuOH fraction obtained from the methanol extract of the aerial parts are lower than that presented by Chemsa et al. [29], since at the concentration of 100 µg/mL it only inhibited AChE and BuChE approximately 16% and 10%, respectively. However, the results obtained from the activity of the newly isolated lignan, rhanteriol (**1**), evaluated on AChE and BuChE, are noteworthy. Specifically, compound **1** showed inhibitory activity against BuChE comparable to that of galantamine, a known BuChE inhibitor used as a standard (IC_50_ 10.41 ± 0.62 µM for compound **1** and 11.63 ± 1.87 µM for galantamine). Moreover, the evaluated activity was higher than lignans isolated from other Tunisian species (*Citharexylum spinosum* L.) since, in this case, the isolated lignans, plucheoside D_1_ and D_2_, possessed an IC_50_ of 98.14 ± 2.72 and 71.28 ± 2.82 µM, respectively [20]. On the other hand, rhanteriol exhibited only weak inhibition of AChE (21% of inhibition at 100 µM). The differences between the rhanteriol inhibition levesl against AChE and BuChE may be explained by considering the results from the molecular-docking analysis. It was indeed seen that AChE possesses a more constricted entrance to the active site pocket than BuChE, thereby contrasting with the entrance of rhanteriol, due to steric hindrance. This is in line with the knowledge that, although AChE is structurally similar to BuChE, six of the fourteen amino acids present on the AChE active site gorge are substituted in BuChE by aliphatic residues. Specifically, F288 and F290 substitution in AChE with smaller L286 and V288 in BuChE determines the formation of a deeper gorge in BuChE, leading to the possibility of BuChE interacting and hydrolyzing a broader range of inhibitors and substrates than AChE [30]. It is indeed known that many inhibitors of AChE and BuChE exert their activity to different degrees [31]. 

Several epidemiological studies suggest a correlation between Alzheimer’s disease and type 2 diabetes mellitus. Glucose uptake from the surrounding environment is impaired in the brains of people with Alzheimer’s disease, since this condition seems to result from brain insulin-resistance [32]. To date, no studies have been reported on the *R. suaveleons* effect in inhibiting the two main enzymes involved in carbohydrate digestion, α-amylase and α-glucosidase. For this reason, in this investigation, it was questioned whether the BuOH extract and rhanteriol could affect the activity of these enzymes, which are known for being therapeutic targets of hypoglycemic drugs such as acarbose. The BuOH extract was shown to inhibit *α*-amylase at a concentration lower than the acarbose used as the standard (IC_50_ 1.47 ± 0.30 and 3.64 ± 0.07 µg/mL, respectively), indicating that the extract is more active. Moreover, the inhibitory activity against α-amylase evaluated for the *R. suaveleons* BuOH fraction was higher than that of *R. adpressum* [33]. In the latter case, the effect of the harvesting time on *R. adpressum* bioactivity was investigated, demonstrating an IC_50_ vs. α-amylase ranging from 1370 ± 0.07 µg/mL to 2710 ± 0.26 µg/mL [33]. However, this difference in bioactivity between the two species may be due to the different extractive solvents used, as MeOH was used for *R. suaveleons*, while acetone and water (70:30) were used for *R. adpressum*, which may result in extracts with a different phytocomplex [34]. This hypothesis is confirmed by the fact that, contrary to *R. adpressum* [33], *R. suaveleons* had no inhibitory activity against α-glucosidase. Moreover, the results obtained on the bioactivity against α-glucosidase are inconsistent if those regarding the bioactivity of rhanteriol are taken into account. It was indeed seen that this new lignan could inhibit both α-amylase and α-glucosidase, with an IC_50_ of 46.42 ± 3.25 and 26.76 ± 3.29 µM, respectively. In particular, for α-glucosidase, the new lignan exerts an inhibition higher than that observed for the acarbose used as the standard (IC_50_ 241.32 ± 4.49 µM) and other lignans such as patulinone A, B, and C, isolated from *Melicope patulinervia* (Merr. & Chun) Huang (IC_50_ ranging from 41.68 ± 3.54 to 95.77 ± 3.63 µM) [34]. Hence, it is possible that other active molecules present in the *R. suaveleons* BuOH fraction may contrast with the α-glucosidase inhibitory activity of rhanteriol. Corroborating the rhanteriol inhibitory effect against α-amylase and α-glucosidase, are the data from the molecular-docking investigation. Notably, in both enzymes, rhanteriol overlapped with the central part of the oligosaccharide analogue of acarbose; however, in the case of alpha-amylase, hydrogen bonds were more frequently formed with the enzyme’s catalytic residues, whereas in the case of α-glucosidase, rhanteriol realized sandwich π-π interactions in the binding pocket. These π-π interactions may be of similar stability to the hydrogen bonds that rhanteriol forms with α-amylase, but stronger, which explains the greater inhibitory activity against α-glucosidase than against α-amylase. 

Polyphenols are known as donors and acceptors of hydrogen bonds, characterized by a hydrophobic nature and flexible backbone. All these features make them good enzyme inhibitors, and certainly, the chemical structure influences the biological activity. For example, caffeic acid and chlorogenic acid, two hydroxycinnamic acids widespread in nature, both reported potential hypoglycemic activity in vitro, but the substitution of an OH group for quinic acid in chlorogenic acid negatively affected inhibitory activity against *α*-amylase and *α*-glucosidase. Quinic acid probably causes steric hindrance in the active sites of both enzymes [35]. It is possible to observe these effects also in the case of flavonoids. Flavan-3-ols, flavonones, anthocyanidins, and flavonols are the most favorable for inhibiting digestive enzymes, due to the presence of a C2=C3 double bond in a C-ring. In particular, flavones and flavonols with a 4-oxo group and OH groups in position 3 (ring C), 7 (ring A), 4′, and 5′ (ring B) are particularly important. Glycosylation negatively affects the activity; for example, luteolin-7-*O*-*β* glucoside and luteolin 4′-*O*-*β*-glucoside reported an IC_50_ higher than that of luteolin [35]. Phenolic compounds exert neuroprotective effects through the active binding sites of AChE and BuChE. Hydroxyl groups are also important for inhibiting cholinesterase enzymes, and free OH groups are more potent than glycosylated ones [36]. Lignan from *R. suaveleons* as well as *S. chinensis* (gomisins and schisandrol B) exhibited remarkable enzyme inhibitory activity, due to the aromatic ring and hydroxyl groups that could directly interact with the active sites of enzymes [7]. In line with findings on other inhibitors, the in silico analysis performed in the present investigation results in a inhibition of α-amylase and α-glucosidase comparable with those of the standard used in this study (acarbose), and BuChE by rhanteriol, suggesting that hydrogen-bond formation and π-π interactions with the catalytic site of these enzymes may involve hydroxyl groups and aromatic rings.

The data obtained indicate that the isolated and characterized novel lignan rhanteriol might represent a promising inhibitor of *α*-amylase, *α*-glucosidase, and BuChE, demonstrating its potential in developing new drugs that might represent future treatments for type 2 diabetes mellitus and neurodegenerative disorders. However, these are preliminary results that need to be further corroborated by more specific in vitro and in vivo tests.

## 4. Materials and Methods

### 4.1. Equipment

Optical rotations were measured on an Atago AP-300 digital polarimeter with a 1 dm microcell and a sodium lamp (589 nm). NMR data were recorded on a Bruker DRX-600 spectrometer at 300 K (Bruker BioSpinGmBH, Rheinstetten, Germany) equipped with a Bruker 5 mm TCI Cryoprobe at 300 K. All 2D NMR spectra were acquired from MeOH-d4 (99.95%, Sigma-Aldrich, Milano, Italy), and standard pulse-sequences and phase cycling were used for the DQF-COSY, HSQC, and HMBC spectra. The NMR data were processed using TOPSPIN 3.2 software (Bruker Biospin, Rheinstetten, Germany). HRESIMS data were obtained on a Q Exactive Plus mass spectrometer, Orbitrap-based FT-MS system, equipped with an ESI source (Thermo Fischer Scientific Inc., Bremen, Germany). Column chromatography was performed over Sephadex LH-20 (Pharmacia). RP-HPLC separations were carried out using a Shimadzu LC-8A series pumping-system equipped with a Shimadzu RID-10A refractive index detector and Shimadzu injector (Shimadzu Corporation, Kyoto, Japan) on a C 18 μ-Bondapak column (30 mm × 7.8 mm, 10 µm, Waters-Milford, Milford, MA, USA). TLC separations were conducted using silica gel 60 F 254 (0.20 mm thickness) plates (Merck, Germany) and Ce(SO_4_)_2_/H_2_SO_4_ as spray reagent (Sigma-Aldrich, Milano, Italy).

### 4.2. Plant Material

The aerial parts of *Rhanterium sualeolens* were collected in May 2016. The plant was identified by Prof. Rebbas Khellaf (University of M’sila, M’Sila, Algeria). An authenticated voucher specimen, with the identification number RHA16SU was deposited at the herbarium of the VARENBIOMOL research unit, University Mentouri of Constantine, Algeria.

### 4.3. Extraction and Isolation

The powdered, dried aerial-parts of *R. suaveolens* (350 g) were extracted with *n*-hexane, CHCl_3_, and MeOH (3 × 1.5 L), to give 2.5 g, 5.6 g, and 13.8 g of the respective extract. The MeOH extract (10 g) was partitioned between *n*-BuOH and H_2_O to give 4.2 g of *n*-BuOH residue. Sephadex LH-20 column chromatography (5 cm × 100 cm) was employed to separate the *n*-BuOH soluble fraction (3.0 g), using as eluent MeOH, at a flow rate of 1.5 mL/min, collecting fractions of 10 mL grouped into seven main fractions (A-G). Fractions F and G gave pure cholorogenic acid (**8**), (18 mg) and 3,4-dicaffeoylquinic acid (**7)**, (20 mg), respectively [35].

Fraction C (270 mg) was purified with RP-HPLC with MeOH-H_2_O (3:2) as eluent to yield compound **1** (4.5 mg, *t*_R_ 40 min), pinoresinol (**2)** (1.0 mg, *t*_R_ 38 min) and coumaric acid (**3**) (5.0 mg, *t*_R_ 15 min). Fraction D (230 mg) was subjected to RP-HPLC with MeOH-H_2_O (4:1) to yield compound **2** (4.0 mg, *t*_R_ 45 min) and caffeic acid (**4**) (4.0 mg, *t*_R_ 22 min). Fraction E (246 mg) was chromatographed over RP-HPLC with MeOH-H_2_O (1:1) to obtain luteolin (**5)** (4.2 mg, *t*_R_ 23 min), quercetin (**6**) (2.0 mg, *t*_R_ 18 min), 4,5-caffeoylquinic-acid (**7**) (2.5 mg, *t*_R_ 35 min) [35]. 

Compound **1,** yellow amorphous powder; ${\left[\alpha \right]}_{D}^{25}$ -33 (*c* 1.0, MeOH); UV (MeOH) λmax (log ε) 235 (4.23), 259 (4.13), 334 (3.85) nm; ^1^H and ^13^C NMR, see Table 1; HRESIMS m/z 487.1717 [M + Na]^+^ (calcd for C_27_H_29_O_7_ 487.1733) and 465.1887 [M + H]^+^.

### 4.4. α-Amylase Inhibition Assay

As reported by Faraone, et al. [36] different doses of each sample were mixed with α-amylase solution (50 μL, 5 U/mL) in phosphate buffer (pH 6.9 with 6 mM sodium chloride) in a 96-well microplate, and incubated for 10 min at 37 °C. Similarly, a blank was prepared by adding sample solution to all reaction reagents without enzyme solution. Subsequently, starch solution (100 μL, 0.1%), the reaction substrate, was added and incubated for 10 min at 37 °C. After that, the reaction was stopped with the addition of HCl (25 μL, 0.1 M), and, successively, the iodine-potassium iodide (KI/I_2_ 0.5 mM) solution (100 μL) was added. The absorbance was read at 630 nm for 10 min. Results are expressed as IC_50_ values (mg/mL for extracts and µM for pure compound) using GraphPad Prism 8 Software (San Diego, CA, USA). The test was carried out in triplicate.

### 4.5. α-Glucosidase Inhibition Assay

The ability of the extracts/pure compound to inhibit the α-glucosidase enzyme was evaluated following a previously described method [36]. Briefly, different doses of each sample were incubated with *α*-glucosidase solution (40 μL, 0.1 U/mL) in phosphate buffer (50 μL, 0.1 M, pH 7) for 10 min. Then, 4-nitrophenyl *α*-D-glucopyranoside (40 μL, 0.5 mM) was added. After 15 min, the reaction was stopped by adding sodium carbonate solution (100 μL, 0.2 M). The absorbance was immediately measured at 405 nm. Results are expressed as IC_50_ values (mg/mL for extracts and µM for pure compound), using GraphPad Prism 8 Software (San Diego, CA, USA). The assay was carried out in triplicate.

### 4.6. Cholinesterase Inhibition Assay 

The inhibition of AChE and BuChE was evaluated using Ellman’s method, as previously reported [37]. Briefly, extracts and pure compound were incubated with AChE or BuChE (25 µL, 0.05 U/mL), DTNB (125 µL, 3 mM) and buffer B (25 µL, 50 mM Tris–HCl, pH 8 containing 0.1% BSA) for 10 min at 37 °C. Then, the substrate of the reaction, the acetylthiocholine iodide or butyrylthiocholine chloride (25 µL, 5 mM), was added, and the absorbance was measured at 405 nm, after 10 min. Galanthamine was used as the standard in both assays. Results are expressed as IC_50_ values (mg/mL for extracts and µM for pure compound), using GraphPad Prism 8 Software (San Diego, CA, USA). The test was carried out in triplicate.

### 4.7. Cell Culture 

HeLa (cervical carcinoma), Jurkat (T-cell leukemia), and U937 (monocytic leukemia) cell lines were obtained from the American Type Cell Culture (ATCC) (Rockville, MD, USA). Cells were maintained in DMEM (HeLa) or RPMI 1640 (Jurkat and U937), supplemented with 10% FBS, 100 mg/L streptomycin and penicillin 100 IU/mL at 37 °C in a humidified atmosphere of 5% CO_2_. To ensure logarithmic growth, cells were subcultured every two days. Stock solutions (50 mM) of purified compound in DMSO were stored in the dark at 4 °C. Appropriate dilutions were prepared in a culture medium immediately prior to use. In all experiments, the final concentration of DMSO did not exceed 0.15% (*v*/*v*). 

Epidermal keratinocytes (HaCaT) were cultured in Dulbecco’s Modified Eagle’s Medium containing 10% fetal bovine serum, supplemented with 100 U/mL each of penicillin and streptomycin, and 2 mM/L glutamine, and grown at 37 °C under 5% CO_2_ air humidified atmosphere. Human leukemia cell lines (Jurkat) were maintained in RPMI medium supplemented with 10% FBS, 2 mM l-glutamine, 100 U/mL penicillin, and 100 mg/mL streptomycin at 37 °C, in 5% CO_2_ [26]. 

### 4.8. Cell Viability 

Cell viability was evaluated using a colorimetric assay based on an MTT ([3-(4,5-dimethylthiazol-2-yl)-2,5-diphenyltetrazolium bromide]) assay, in order to compare the effect of potentially cytotoxic substances with a control condition. Briefly, cells were plated in 96-well tissue culture plates (3.5 × 103 cells/well) and after 24 h, the medium was replaced with a fresh one alone or one containing serial dilutions of compound **1** (100, 50, 25 µM), and incubation was performed for 48 h. Staurosporin 0.2 µM was used as a positive control. At the end of treatment, an amount of 25 μL of MTT (5 mg/mL) was added to each well, and cells were incubated for an additional 3 h, to allow the formation of purple formazan precipitate; then, 100 μL of a solution containing 50% (*v*/*v*) N,N-dimethylformamide, 20% (*w*/*v*) SDS with an adjusted pH of 4.5 were added. The optical density (OD) of each well was measured with a microplate spectrophotometer (Multiskan Spectrum Thermo Electron Corporation reader, Thermo Fisher Scientific, Waltham, Massachusetts, United States) equipped with a 620 nm filter. Cell vitality was calculated as % vitality = 100 × (OD treated/OD DMSO) [38]. 

### 4.9. In Silico Molecular Docking

Molecular docking was performed by AutoDock Vina [39] with conformationally flexible rhanteriol in the human protein structures of pancreatic α-amylase (PDB ID: 1XD0) [40] and small intestine α-glucosidase (PDB ID: 3TOP) [41], as well as in a homology model of human BuChE [42]. The side chains of the residues in the substrate binding pocket of each protein were chosen to have conformational flexibility during the docking: α-amylase (W58, W59, Y62, Q63, D96, V98, H101, Y151, L162, T163, L165, R195, D197, K200, H201, D233, I235, E240, F256, N298, H299, D300, H305 and R337), α-glucosidase (K1156, D1157, Q1158, P1159, Y1167, Y1251, D1279, I1280, Q1286, I1315, D1317, W1355, W1369, Q1372, K1377, W1418, D1420, M1421, E1423, S1425, F1427, K1460, R1510, W1523, D1526, T1528, D1555, F1559, F1560, Q1561, R1582, H1584, T1586 and I1587) and BuChE (Q95, N96, D98, W110, W140, Y142, F146, Q147, T148, T150, E225, S226, S252, W259, V305, E353, F357, Y360, I426, H466, E469 and I470). The docking results were analyzed using PyMOL and PISA [43]. 

### 4.10. Statistical Analysis 

Data are expressed as mean ± standard deviation (Mean ± SD). Statistical analysis was performed by analysis of variance (one-way ANOVA) followed by Tukey’s test, using GraphPad Prism 8 Software, Inc. (San Diego, CA, USA), and a *p*-value of 0.05 or less was considered as statistically significant. All measurements were performed using SPECTROstarNano (BMG Labtech, Ortenberg, Germany) [36].

## 5. Conclusions

The BuOH fraction of the *Rhanterium suaveolens* aerial-part methanolic extract was investigated for its ability in inhibiting enzymes involved in neurodegeneration and, for the first time, in carbohydrate digestion. Furthermore, its characterization allowed the identification of seven known compounds and the isolation of one new lignan. This new compound was identified using spectroscopic data as rhanteriol, and its bioactivity was evaluated for the first time, showing a promising inhibition of *α*-amylase, *α*-glucosidase, and BuChE. Computational studies have also provided significant features concerning the binding of the new lignan to the active site, thus confirming rhanteriol’s ability to inhibit enzymes involved in carbohydrate digestion and neurodegeneration. Therefore, it is possible to assert that the aerial part of *Rhanterium suaveolens* could represent a source of active molecules such as rhanteriol, usable in the development of future treatments for preventing or treating type 2 diabetes mellitus and neurodegenerative disorders. However, it is important to take into consideration the fact that the spectrophotometric assays used for evaluating the biological activities of either the BuOH extract fraction or the isolated compound are not directly related to real actions in more complex systems such as cells or in vivo models. Hence, to clarify the potential of *Rhanterium suaveolens* in counteracting type 2 diabetes and neurodegenerative disease, further investigations will be performed through in vitro and in vivo tests. 

## Figures and Tables

**Table 1:** NMR spectroscopic data of compound **1** (CD_3_OD, 600 MHz) ^a^.

Position		1 ^a^	HMBC
	δ_H_	δ_C_	
1		133.0	
2	6.82 d (1.5)	111.1	3, 4, 6,
3		149.0	
4		147.6	
5	6.74 d (8.0)	116.3	1, 3
6	6.69 dd (8.0, 1.8)	122.1	2, 7
7a	2.95 dd (12.5, 5.4)	34.3	1, 2, 6, 8, 8′
7b	2.64 dd (12.5, 10.3)	-	1, 2, 6, 8, 8′
8	2.90 m	44.3	
9a	4.10 dd (8.8, 6.5)	72.5	7, 8, 7′, 8′
9b	3.80 °		7, 8, 7′, 8′
-OMe	3.84 s	56.3	3
1′		134.9	
2′	6.94 (2.0)	111.2	1′, 4′, 6′, 7′
3′		149.0	
4′		147.5	
5′	6.77 d (8.0)	116.1	1′, 3′
6′	6.85 dd (8.0, 2.0)	120.3	2′, 4′, 7′
7′	4.87 °	85.4	1′, 2′, 6′, 9′
8′	2.78 m	50.5	7′, 8, 9′
9a’	4.67 dd (11.0, 6.2)	64.6	7′, 8, 8′, 7″
9b’	4.47 dd (11.0, 8.0)		7′, 8, 8′, 7″
1″		134.9	
2″	7.82 m	130.5	1″, 6″, 7″
3″	7.44 t (8.0)	129.5	1″, 5″
4″	7.60 br t (8.0)	134.2	
5″	7.44 t (8.0)	129.5	1″, 5″
6″	7.82 m	130.5	1″, 6″, 7″
7″		167.8	
-OMe	3.78 s	56.4	

^a^ Chemical shifts are given in ppm; assignments were confirmed by Homonuclear Correlated Spectroscopy (H-H-COSY), Heteronuclear Single Quantum Coherence (HSQC) and Heteronuclear Multiple Bond Correlation (HMBC) experiments. ° Overlapped signal.

## Data Availability

Not applicable.

## Supplementary Materials

The following supporting information can be downloaded at https://www.mdpi.com/article/10.3390/plants12020301/s1, Figure S1: ^1^H NMR spectrum of compound **1** (CD_3_OD, 600 MHz); Figure S2: ^13^C NMR spectrum of compound **1** (CD_3_OD, 150 MHz); Figure S3: COSY spectrum of compound **1** (CD_3_OD, 600 MHz); Figure S4: HSQC spectrum of compound **1** (CD_3_OD, 600 MHz); Figure S5: HMBC spectrum of compound **1** (CD_3_OD, 600 MHz); Figure S6: HRESIMS of compound **1**.

## Sample Availability

Samples of the compounds are available from the authors.
