# Supplementary material for: Rhanteriol, a New Rhanterium suaveolens Desf. Lignan with Pharmacological Potential as an Inhibitor of Enzymes Involved in Neurodegeneration and Type 2 Diabetes
Source: Plants (Basel). 2023 Jan 9;12(2):301. doi: 10.3390/plants12020301 (PMC9865629; doi:10.3390/plants12020301)

Supporting Information

Rhanterіol, a new *Rhanterium suaveolens* Desf. lignan with pharmacological potential as potent enzyme inhibitor

Soumia Belaabed [1], Ayoub Khalfaoui [1], Valentina Parisi [2,3], Valentina Santoro[2], Daniela Russo [4], Maria Ponticelli [4*], Magnus Monné [4], Khellaf Rebbas [5], Luigi Milella [4*], and Giuliana Donadio [2].

[1] Department of chemistry, Research unit, Development of natural resources, Bioactive molecules, Physicochemical and biological analysis, University Mentouri, Route Ain ElBey 25000 Constantine, Algeria

[2] Dipartimento di Farmacia, Università degli Studi di Salerno, via Giovanni Paolo II 132, 84084 Fisciano (SA), Italy

[3.] PhD Program in Drug Discovery and Development, Department of Pharmacy, University of Salerno, Via Giovanni Paolo II 132, 84084, Fisciano, Salerno, Italy.

[4] Dipartimento di Scienze, Università degli Studi della Basilicata, Viale dell'Ateneo Lucano 10, 85100 Potenza, Italy

[5] Natural and Life Sciences Department, Mohamed Boudiaf University, M'sila, Algeria

* Correspondence: luigi.milella@unibas.it (L.M.); maria.ponticelli@unibas.it (M.P.) Tel.: +39 0971 205525

Table of Contents

Figure S1. ^{1}H NMR spectrum of compound **1** (CD_3OD, 600 MHz)

Figure S2. ^{13}C NMR spectrum of compound **1** (CD_3OD, 150 MHz)

Figure S3. COSY spectrum of compound **1** (CD_3OD, 600 MHz)

Figure S4. HSQC spectrum of compound **1** (CD_3OD, 600 MHz)

Figure S5. HMBC spectrum of compound **1** (CD_3OD, 600 MHz)

Figure S6. HRESIMS of compound **1**

Figure S1. ^{1}H NMR spectrum of compound **1** (CD_3OD, 600 MHz)

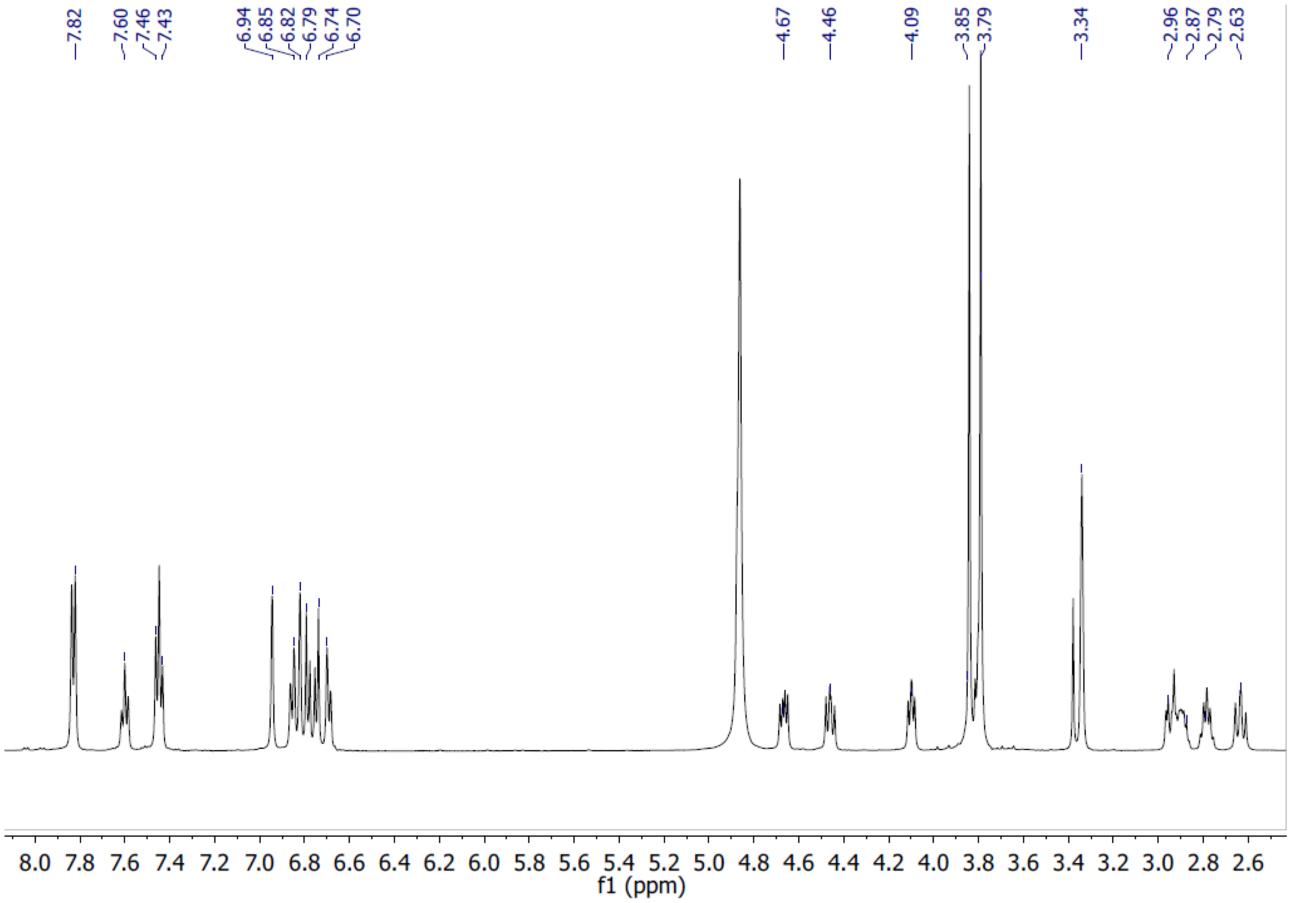

Figure S2. ^{13}C NMR spectrum of compound **1** (CD_3OD, 150 MHz)

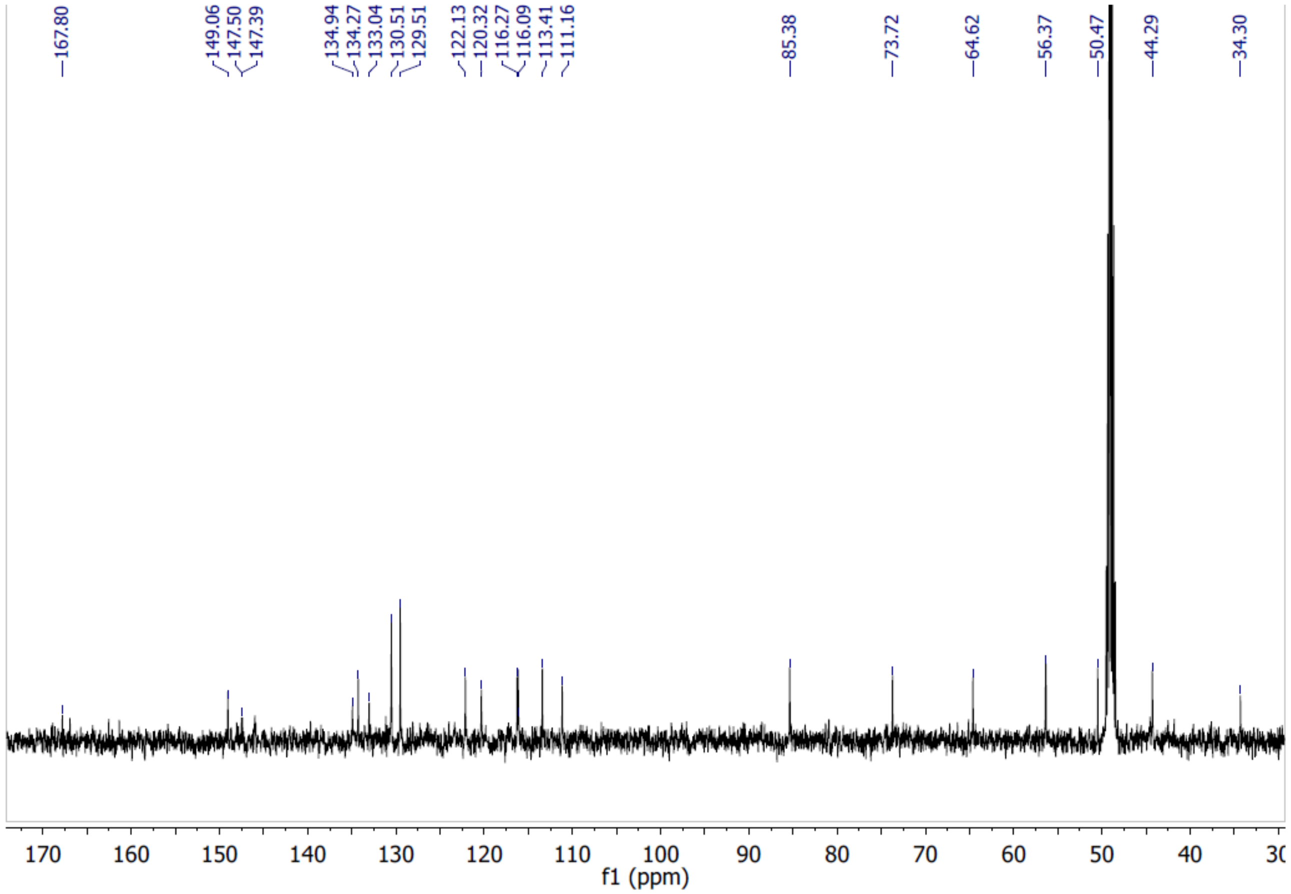

Figure S3. COSY spectrum of compound **1** (CD_3OD, 600 MHz)

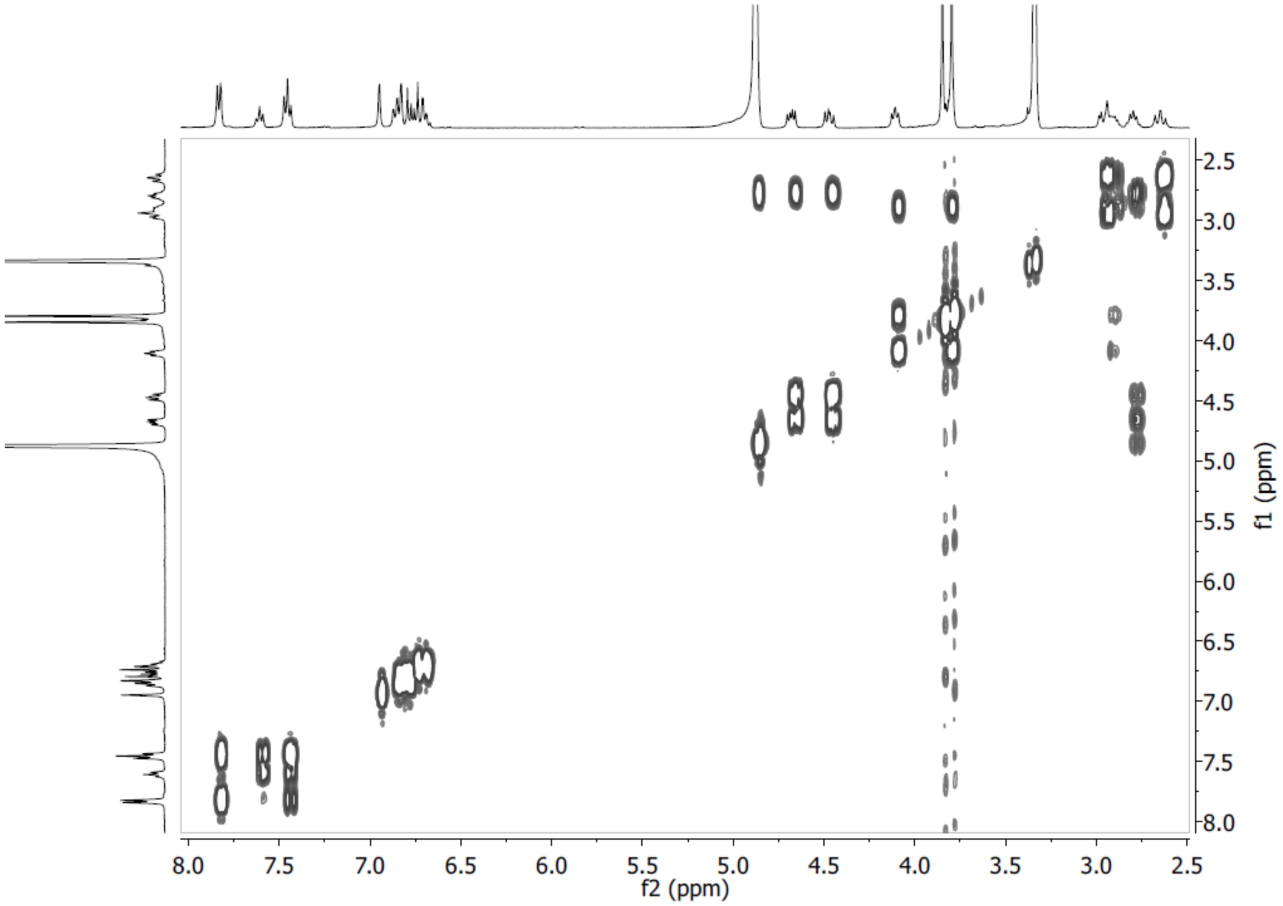

Figure S4. HSQC spectrum of compound **1** (CD_3OD, 600 MHz)

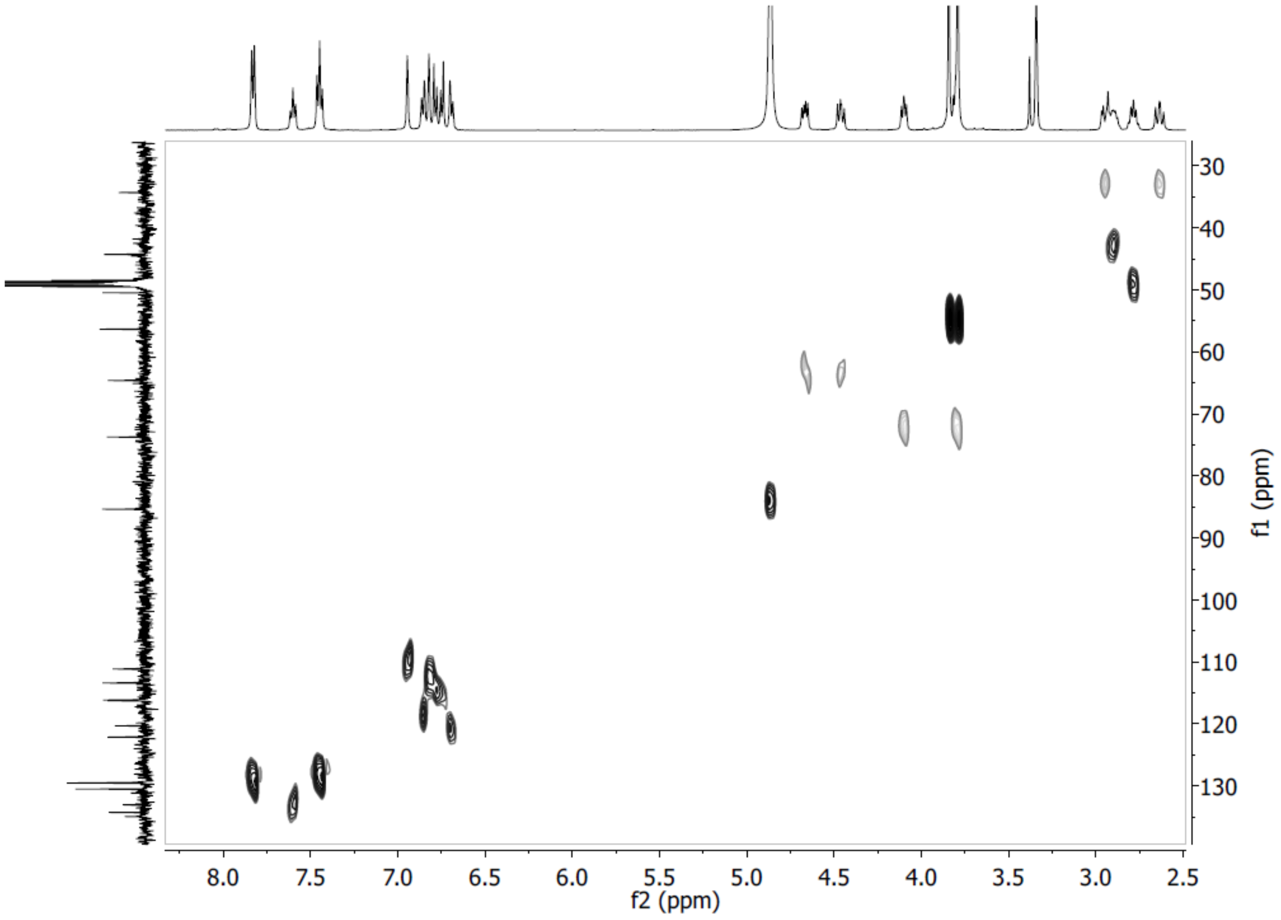

Figure S5. HMBC spectrum of compound **1** (CD_3OD, 600 MHz)

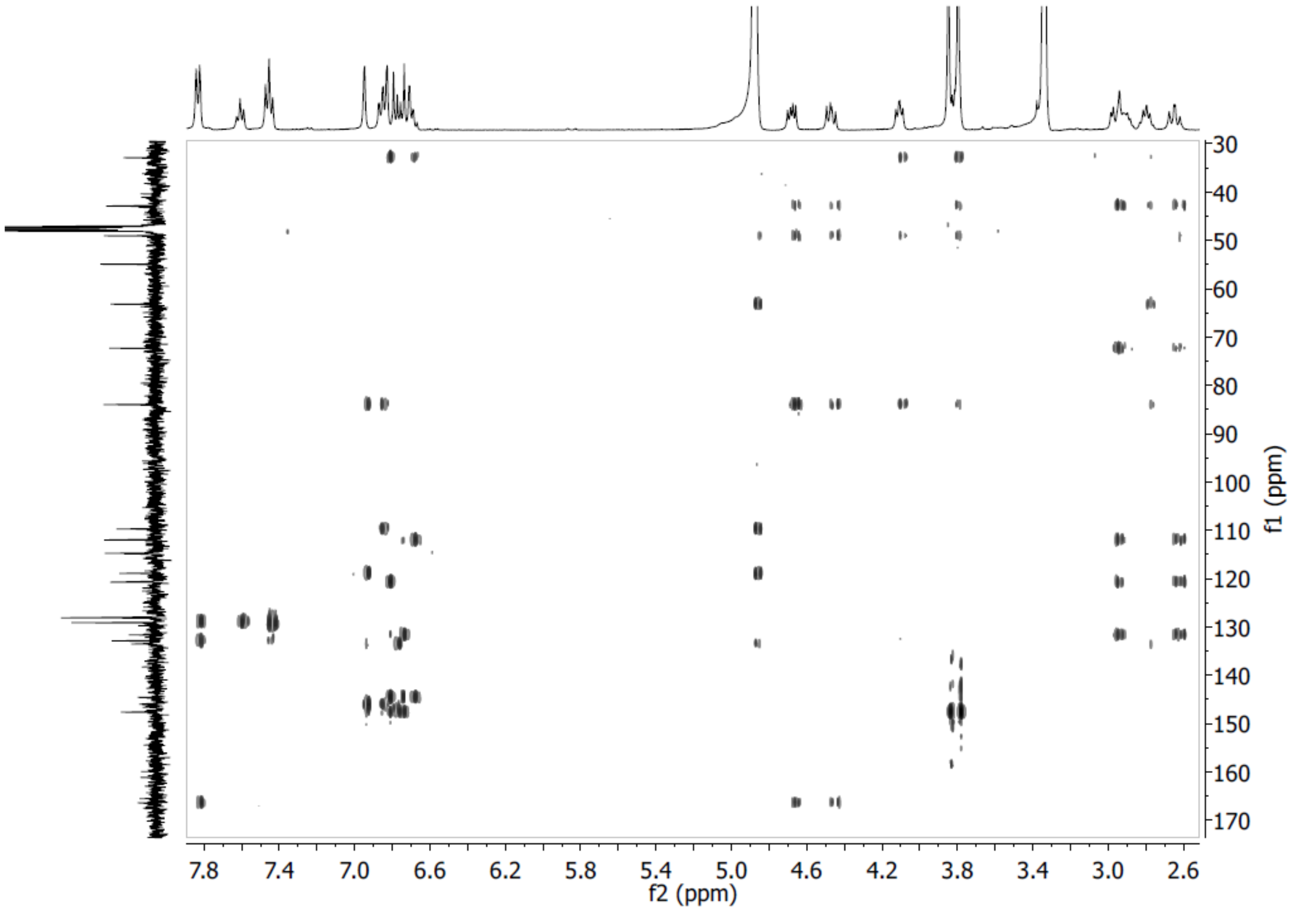

Figure S6. HRESIMS of compound **1**

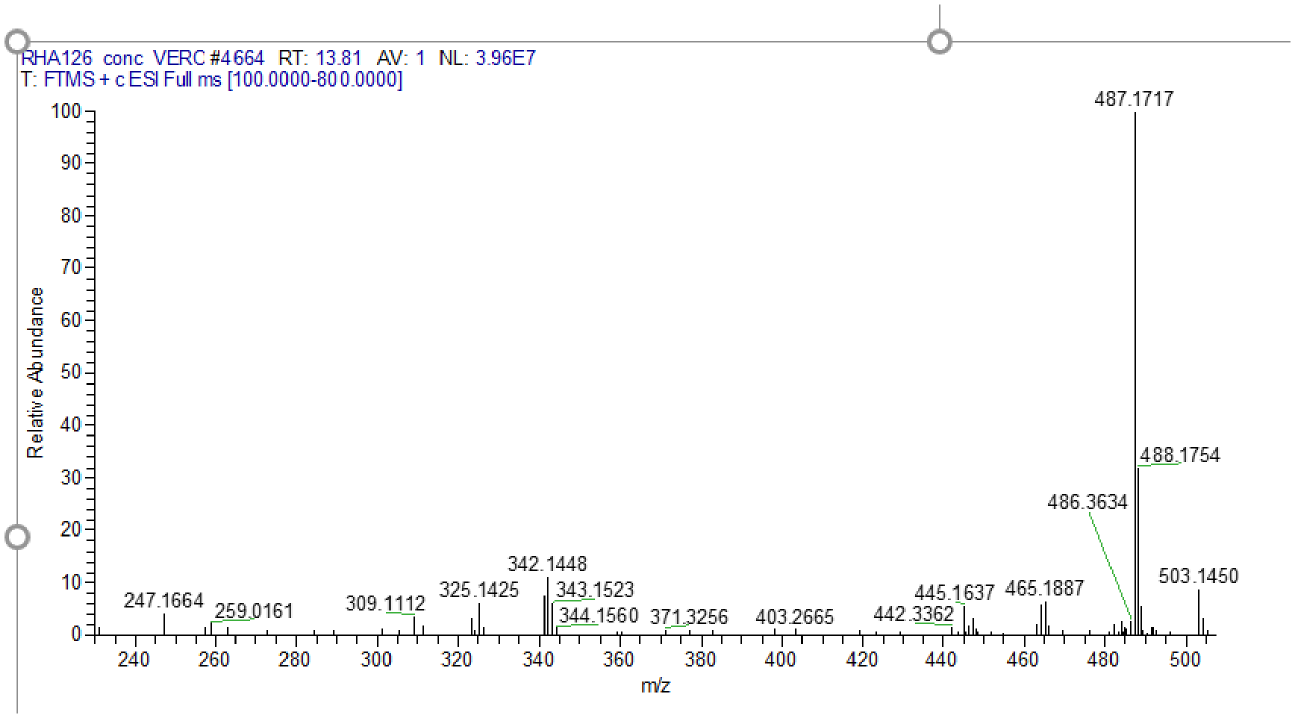

Supplement: Supplementary file 1 [file plants-12-00301-s001.zip › plants-2089601-supplementary.pdf]
